# Solute Carrier Family 1 Member 5 (SLC1A5)-dependent glutamine transport regulates macrophage senescence and efferocytosis during liver regeneration

**DOI:** 10.1186/s43556-026-00526-0

**Published:** 2026-09-01

**Authors:** Haoran Hu, Tao Han, Qiyuan Huang, Ping Gong, Jiangxi Liu, Hao Wang, Xiaowei Ling, Kang Wang, Shuangshuang Liu, Yue Yu, Ting Luo, Feng Cheng, Xiaoxiao Liu

**Affiliations:** 1 Hepatobiliary Center, The First Affiliated Hospital with Nanjing Medical University, Research Unit of Liver Transplantation and Transplant Immunology, Key Laboratory of Liver Transplantation, Chinese Academy of Medical Sciences, Key Laboratory of Hepatobiliary Tumors, National Health Commission, Jiangsu Provincial Medical Innovation Center, Jiangsu Provincial Medical Key Laboratory, Nanjing, 210029 China; 2 Department of Radiation Oncology, Cancer Center, Affiliated Hospital of Xuzhou Medical University, Cancer Institute, Xuzhou Medical University, Xuzhou, Jiangsu 221000 China; 3 Institute of Breast Health Medicine, Breast Center, Department of Medical Oncology, Cancer Center, West China Hospital, Sichuan University, Chengdu, Sichuan 610041 China; 4 Department of General Surgery, People’s Hospiltal of Jinhu, Huaian, Jiangsu 213200 China; 5 Jiangsu Key Laboratory of Organ Transplantation and Transplant Immunology and Hepatobiliary Center, Affiliated Hospital of Xuzhou Medical University, Xuzhou, Jiangsu 221000 China

**Keywords:** Liver regeneration, Macrophage, SLC1A5, Cell senescence, Efferocytosis

## Abstract

The liver possesses an extraordinary capacity to regenerate after injury or surgical resection, a process highly dependent on the coordinated orchestration of the immune microenvironment. Although macrophages are recognized as pivotal coordinators of hepatic tissue repair, the precise checkpoints governing their functional transitions during regeneration remain elusive. Here, we identify the glutamine transporter SLC1A5 (Solute Carrier Family 1 Member 5) as a critical metabolic gatekeeper of macrophage function during liver regeneration. Using a mouse model of partial hepatectomy, we show that SLC1A5 is markedly upregulated in monocyte-derived macrophages at the peak of regeneration. Myeloid specific deletion of *Slc1a5* (*Slc*1*a*5^fl/fl^*Lyz*2^cre^) severely impairs hepatocyte proliferation and diminishes the expression of macrophage derived regenerative factors. Mechanistically, *Slc1a5* deficiency depletes intracellular glutamine, which triggers macrophage senescence and drives a pro-inflammatory phenotype. This senescent state selectively downregulates *Gas6* (Growth Arrest Specific 6), a crucial bridging ligand for efferocytosis, thereby impairing apoptotic cell clearance and exacerbating local inflammation. Strikingly, exogenous *Gas6*, senolytic Quercetin therapy, or in vivo L-glutamine supplementation successfully alleviates macrophage senescence, reinstates Gas6 mediated efferocytosis, and rescues defective liver regeneration. Collectively, our findings reveal a novel ‘*Slc1a5*-glutamine-senescence-efferocytosis’ axis that dictates macrophage driven tissue repair. This study not only uncovers a fundamental immunometabolic mechanism but also highlights glutamine supplementation and senolytics therapy as promising clinically strategies to accelerate liver regeneration.

**Supplementary Information:**

The online version contains supplementary material available at https://doi.org/10.1186/s43556-026-00526-0.

## Introduction

Liver has remarkable regenerative capacity. Human livers can regrow to their original size even after 90% volume resection [1]. This unique regenerative function ensures the maintenance of hepatic and systemic homeostasis [2]. Liver regeneration is tightly orchestrated by metabolic and immune regulation. Reprogramming of metabolic pathways including glucose, lipid and amino acid metabolism regulate liver regeneration in their different ways, which have been reviewed [3]. Hepatocytes serve as the main regenerative cells during liver regeneration, with different immune cells exerting either promotive or inhibitory effects [4]. To date, substantial gaps persist in therapeutic strategies aimed at enhancing liver regeneration; the first-in-class medicine, HRX215, has not yet entered clinical practice [5]. Thus, deciphering the underpinning mechanisms of liver regeneration could help enhance recovery of liver function after resection and facilitate the development of novel therapeutic approaches.

Hepatocytes are the primary drivers of liver regeneration, but this process is tightly regulated by the coordinated crosstalk with various intrahepatic cell types, including hepatic stellate cells, liver sinusoidal endothelial cells, biliary epithelial cells and et al. [6–9] Among these, macrophages have emerged as key regulators due to their remarkable phenotypic plasticity and central role in orchestrating the inflammatory and reparative phases of liver injury. In recent years, the significant role of macrophages in tissue regeneration has been emphasized, including liver [10]. During liver injury, macrophages first amplify inflammation and then, as the cytokine and metabolite milieu shifts, switch to a reparative phenotype. This transition is governed by signaling like *Wnt-β-catenin* and *Foxo3*, which rewire transcriptional programs to terminate inflammation and promote tissue repair [11]. Studying these changes in macrophages phenotypes and functions may provide new insights for strategies to promote liver regeneration.

Macrophage efferocytosis, the rapid, immunologically silent clearance of apoptotic cells, limits sterile inflammation and is required for timely transition to the reparative phase after acute liver injury [12]. Defective macrophage efferocytosis in liver injury because of reduction of phagocytic receptor impaired the clearance of dead cells, whose intracellular contents will cause severe inflammation [13, 14]. It has been demonstrated that efferocytotic macrophages undergo anti-inflammatory reprogramming due to changes in intracellular metabolites such as lipids and tryptophan [15, 16]. However, it remains unclear how macrophage efferocytosis receptor or ligand changes during liver regeneration.

Cellular senescence, a pathological response to DNA damage, oxidative stress or mitochondrial dysfunction also plays vital role in regeneration after liver injury. Different senescent cells perform various functions during different phase of liver regeneration. It has been reported that clearance of senescent macrophage could relieve liver injury and promote repair [17]. Glutamine, an important metabolite, was found suppress senescence in many diseases, like intervertebral disc, by regulating glycose metabolism and autophagy [18]. Its function of regulating macrophage anti-inflammatory reprograming and boosting tissue regeneration is also reported [19, 20]. Recently, macrophage was found able to harness hepatocyte glutamate and convert it into glutamine to boost liver regeneration [21].

Solute Carrier Family 1 Member 5 (SLC1A5, also known as ASCT2), a neutral amino acid transporter who is widely recognized be capable transport glutamine, play vital role in macrophage functional change [22, 23] SLC1A5 sustains glutamine supply to support metabolic pathways like the tricarboxylic acid cycle and nucleotide synthesis, which are required for macrophage polarization and cytokine production [24, 25]. However, despite SLC1A5 established role in macrophage inflammatory phenotype shift, whether SLC1A5 mediates glutamine transport in macrophages during liver regeneration, and how this process intersects with senescence and efferocytosis, two key determinants of macrophage pro-repair function, remains unknown.

In this study, we hypothesized that macrophage SLCA5 deficiency mediated senescence impairs liver regeneration by driving a persistent pro-inflammatory response. To test this, we utilized a mouse model of partial hepatectomy with myeloid specific *Slc1a5* knockout mice and in vitro macrophage assays. We identified a critical role for Slc1a5 mediated glutamine transport in preventing macrophage senescence and sustaining their efferocytotic capacity. Furthermore, our findings reveal a novel metabolic-immune axis wherein Slc1a5 dependent glutamine transport preserves the reparative functions of macrophages. Consequently, targeting Slc1a5 mediated glutamine transport or enhancing macrophage efferocytosis represents a promising therapeutic strategy to accelerate liver regeneration.

## Results

### Upregulation of macrophage *Slc1a5* during liver regeneration

**Fig. 1:**
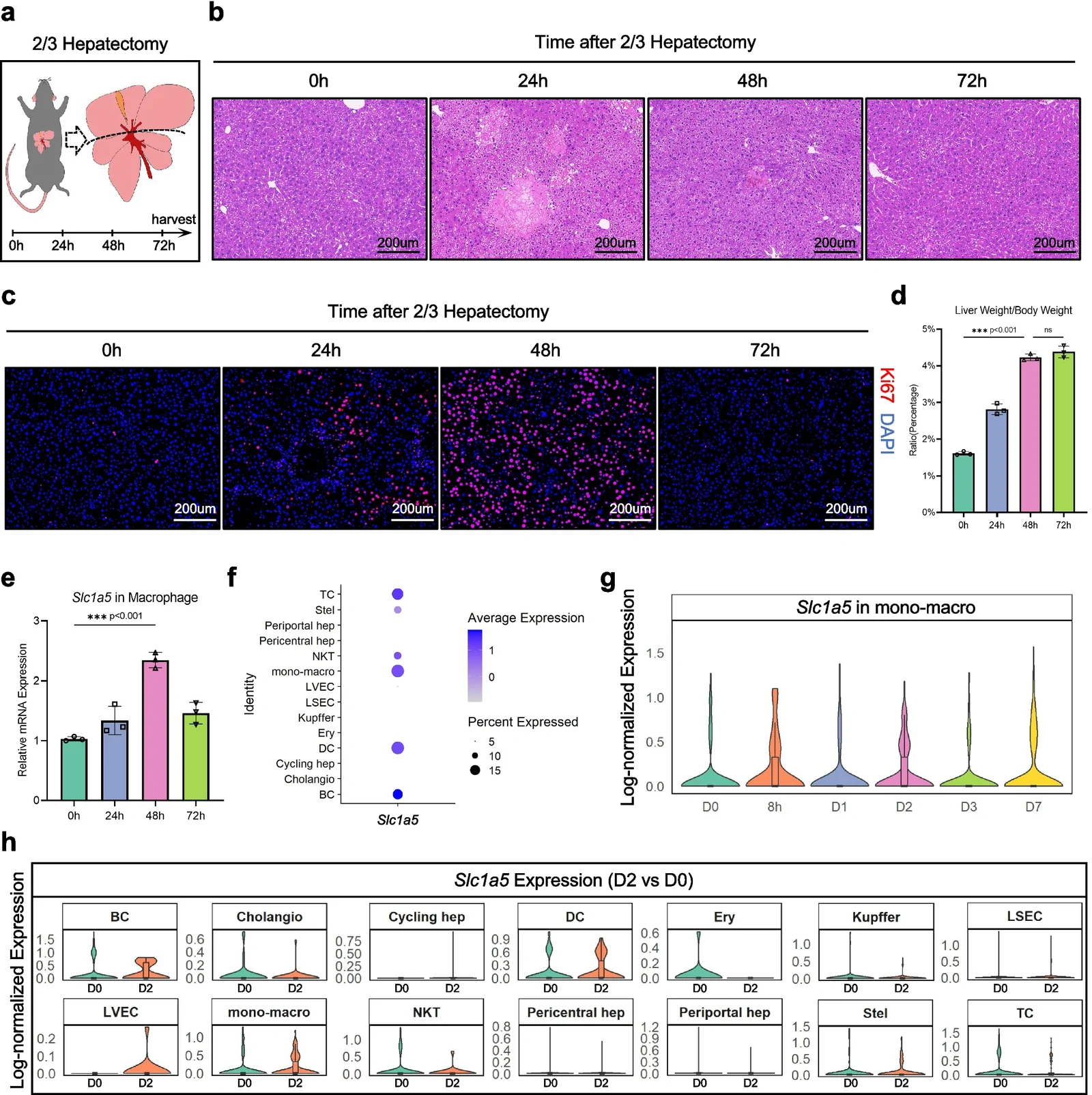
Analysis of *Slc1a5* expression in regenerative liver. **a** Wild type C56BL/6 mice were subjected to 2/3 hepatectomy model as methods described. (*n* = 5/group). **b** Representative H&E staining of liver section in 0, 24, 48 and 72 h after hepatectomy (scale bar: 200 μm). **c** Representative Ki67 staining of liver section in 0, 24, 48 and 72 h after hepatectomy (scale bar: 200 μm). **d** Statistics analysis of liver weight/body weight ratio. **e** Intrahepatic macrophage was extracted as methods described and PCR was used to detect *Slc1a5* expression. Analysis of public scRNA-seq dataset (China National GeneBank Database (CNGBdb): CNP0002310) of mice liver hepatectomy model. **f** Dot Plot showing *Slc1a5* expression in different types of cells. **g** *Slc1a5* expression in mono-macrophage in different time points were shown with Violin Plot. **h** Grouped Violin Plot showing *Slc1a5* change in Day2 vs. Day0 in different types of cells. Statistical significance was defined as *p* < 0.05. (*p < 0.05, **p < 0.01, ***p < 0.001)

SLC1A5 has recently garnered attention due to its role in transporting glutamine, regulating metabolism and immune function, and emerging as a promising target for modulating liver regeneration processes [26, 27]. However, the role of SLC1A5 in macrophages during liver regeneration has not yet been elucidated. Therefore, we first established a classic liver regeneration model in WT mice and collected samples at different time points to investigate changes in liver regeneration and intrahepatic macrophage *Slc1a5* during the process. A 2/3 hepatectomy model was conducted in WT mice and continuous time points (0, 24, 48, 72 h) were included (Fig. 1a). Through H&E staining (Fig. 1b), gradual restoration of liver histology was observed over time. *Ki67* immunofluorescence staining of liver sections showed that intrahepatic proliferating cells gradually increased over time, peaking at 48 h, but significantly decreased at 72 h (Fig. 1c). The liver weight/body weight ratio showed a similar pattern, increasing over time but changing little after 48 h (Fig. 1d). To investigate changes in *Slc1a5* in macrophages, intrahepatic macrophages were isolated and extracted. PCR revealed that *Slc1a5* expression in macrophages increased with the liver regeneration process, peaking at 48 h and decreased at 72 h (Fig. 1e). The above results indicate 48 h post-hepatectomy as the peak of tissue regeneration [28, 29]. coinciding with the peak expression of *Slc1a5* in intrahepatic macrophages. To further verify this inference, public liver regeneration scRNA-seq dataset (China National GeneBank Database (CNGBdb): CNP0002310) from mouse partial hepatectomy model was utilized to analyze the expression of *Slc1a5* in different cell types throughout the course of liver regeneration [30]. It was found that *Slc1a5* was primarily expressed (over 15%) in monocyte-derived macrophages, DC (Dendritic cell), BC (B cell), and TC (T cell) (Fig. 1f). Analysis of *Slc1a5* in mono-macrophages at different time points showed a marked upregulation of *Slc1a5* expression on Day 2 post-hepatectomy compared to Day 0 (Fig. 1.g). Further exploration of *Slc1a5* expression in all cell types at 48 h versus 0 h also revealed significant changes in mono-macro cells (Fig. 1h). The above results further suggest that the upregulation of *Slc1a5* expression in macrophages may play a role in the process of liver regeneration.

### Macrophage *Slc1a5* deficiency impairs liver regeneration

**Fig. 2:**
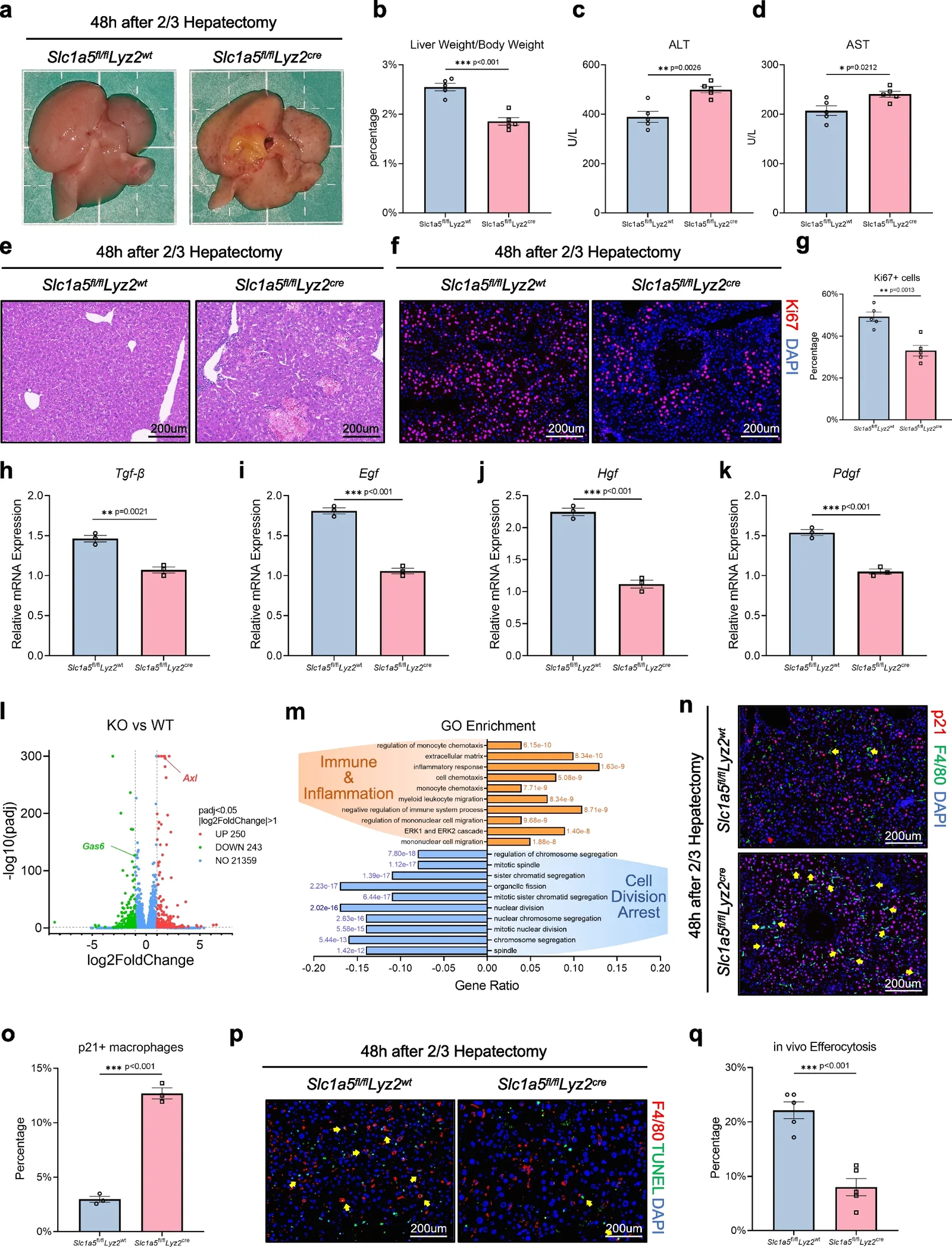
Macrophage *Slc1a5* deficiency impaired liver regeneration in vivo. *Slc1a5*^*fl/fl*^*Lyz2*^*cre*^ mice and *Slc1a5*^*fl/fl*^*Lyz2*^*wt*^ mice were subjected to 2/3 hepatectomy model as methods described. (*n* = 5/group). **a** General view of liver in 48 h after hepatectomy. **b** Statistics analysis of liver weight/body weight ratio. **c**-**d** Statistics analysis of serum ALT and AST. **e** Representative H&E staining of liver section (scale bar: 200 μm). **f** Representative Ki67 staining of liver section (scale bar: 200 μm). **g** Statistics analysis of Ki67 positive cells percentage in liver. **h**–**k** PCR analysis of *Tgf-β*, *Egf*, *Hgf* and *Pdgf* in liver. BMDMs was isolated from *Slc1a5*^*fl/fl*^*Lyz2*^*cre*^ mice and *Slc1a5*^*fl/fl*^*Lyz2*.^*wt*^ mice and processed as methods described. (*n* = 3/group). **l** Volcano plot to show differential gene from RNA-seq. **m** GO enrichment analysis of up-regulated and down-regulated genes. **n** Representative p21 and F4/80 staining of liver section. Double-positive cells are indicated by yellow arrows. (scale bar: 200 μm). **o** Statistics analysis of p21 and F4/80 double positive cells in F4/80 positive cells. **p** Representative TUNEL and F4/80 staining of liver section. Efferocytotic macrophages are indicated by yellow arrows. (scale bar: 200 μm). **q** Statistics analysis of F4/80^+^ cells adjacent to TUNEL^+^ cells in all F4/80^+^ cells to measure in vivo efferocytosis. Statistical significance was defined as *p* < 0.05. (*p < 0.05, **p < 0.01, ***p < 0.001)

To explore the precise role of *Slc1a5* in macrophages during liver regeneration, myeloid-specific *Slc1a5*-deficient mice (*Slc1a5*^*fl/fl*^*Lyz2*^*cre*^) and littermate control (*Slc1a5*^*fl/fl*^*Lyz2*^*wt*^) mice were used to generate 2/3 hepatectomy model. General appearance of liver size and liver weight/body weight ratio showed that macrophage *Slc1a5* deficiency impaired liver regeneration (Fig. 2a-b). HE staining and transaminase ALT&AST also exhibited incomplete tissue repair within the liver and poor recovery of liver function (Fig. 2c-e). Ki-67 staining suggested decrease of regenerative cells in the *Slc1a5* myeloid deficiency liver (Fig. 2f-g). PCR also found that the production of common regenerative factors, *Tgf-β*, *Egf*, *Hgf* and *Pdgf*, [31] reported generated by macrophages has decreased (Fig. 2h-k). These results suggest that *Slc1a5* deficiency in macrophages impairs liver regeneration.

To further investigate the changes in macrophages due to *Slc1a5* deficiency, murine bone marrow derived macrophages (BMDMs) from *Slc1a5*^*fl/fl*^*Lyz2*^*cre*^ and *Slc1a5*^*fl/fl*^*Lyz2*^*wt*^ mice were isolated and cultured until mature for RNA-seq. 250 upregulated genes and 243 downregulated genes were detected (Fig. 2l). Interestingly, *Axl*, A member of the TAM efferocytosis receptor family, was found increased in *Slc1a5* deficient macrophage while *Gas6*, a bridge ligand of efferocytosis, decreased significantly. GO enrichment analysis showed that the up-regulated genes are mainly enriched in inflammatory response while down-regulated genes in multiple cell division-related pathway including nuclear division, nuclear chromosome segregation, mitotic nuclear division among others. (Fig. 2m). Co-staining of F4/80 and p21 in vivo showed that macrophage *Slc1a5* knockout increased senescent macrophages (F4/80^+^ p21^+^ cells) significantly (Fig. 2n-o). Efferocytosis in vivo was also detected by co-staining of F4/80 and TUNEL. F4/80^+^ macrophages immediately adjacent to TUNEL^+^ cells are believed to be undergoing efferocytosis. The proportion of macrophages undergoing efferocytosis in mice was significantly reduced (Fig. 2p-q). Therefore, it is hypothesized that macrophage *Slc1a5* deficiency impairs liver regeneration due to its propensity for senescence and pro-inflammatory phenotype, possibly also linked to reduced efferocytosis.

### Macrophage *Slc1a5* deficiency drives cell senescence and promote inflammation

**Fig. 3:**
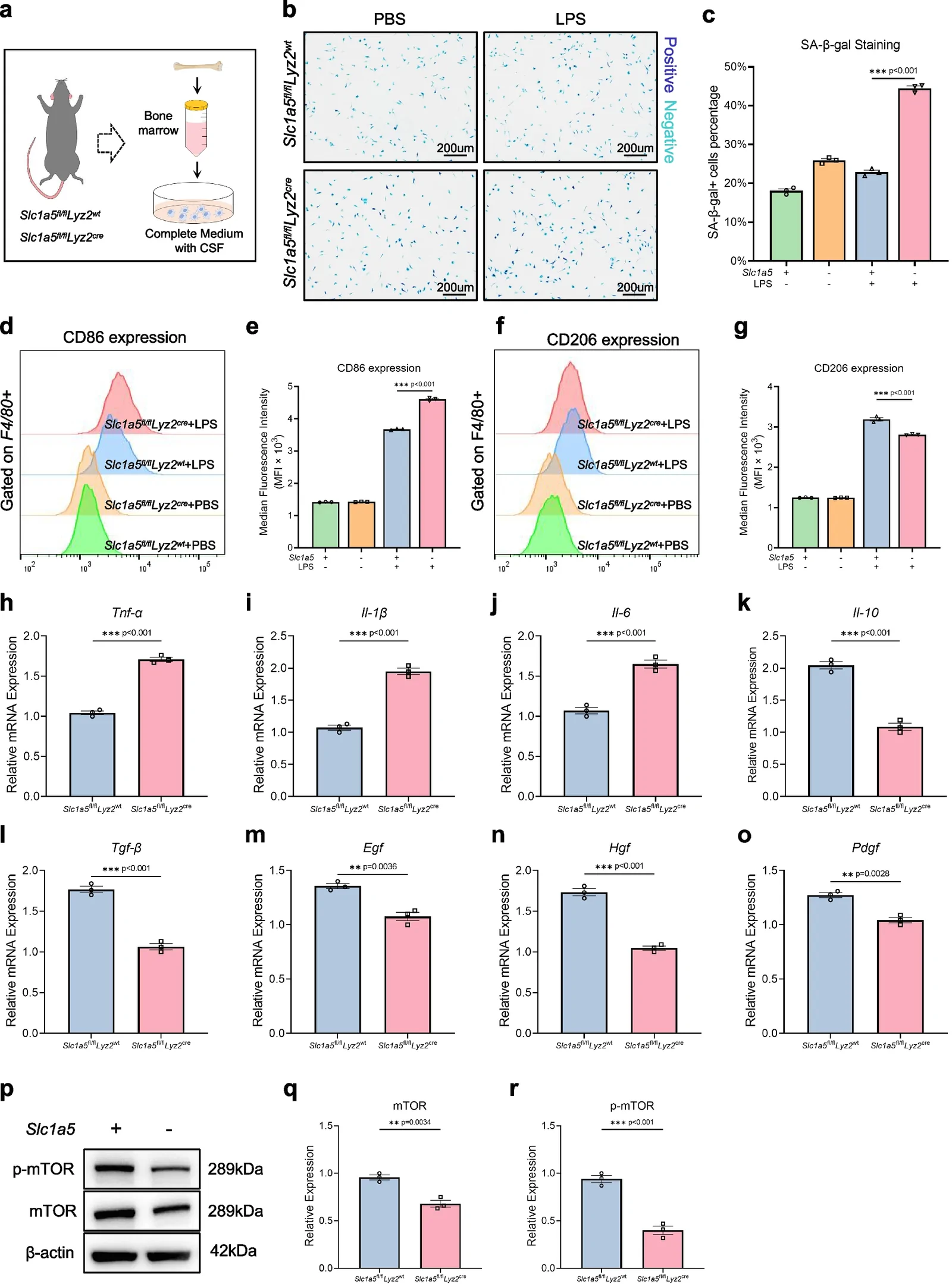
Macrophage *Slc1a5* deficiency induced cell senescence and inflammation. **a** BMDMs was isolated from *Slc1a5*^*fl/fl*^*Lyz2*^*cre*^ mice and *Slc1a5*^*fl/fl*^*Lyz2*.^*wt*^ mice and processed as methods described. (*n* = 3/group). LPS was used to stimulate BMDMs. **b** Representative SA-β-gal staining of BMDMs. **c** Statistics analysis of SA-β-gal positive cells percentage of BMDMs. **d** Flow cytometry analysis of macrophage CD86 expression to measure pro-inflammatory polarization. **e** Statistics analysis of MFI of CD86 in BMDMs. **f** Flow cytometry analysis of macrophage CD206 expression to measure anti-inflammatory polarization. **g** Statistics analysis of MFI of CD206 in BMDMs. **h**–**k** PCR analysis of *Tnf-α*, *Il-1β*, *Il-6* and *Il-10* in LPS treated BMDMs. **l**-**o** PCR analysis of *Tgf-β*, *Egf*, *Hgf* and *Pdgf i*n LPS treated BMDMs. **p** Western Blot analysis of mTOR and p-mTOR expression. **q**-**r** Statistics analysis of mTOR and p-mTOR expression relative to β-actin. Statistical significance was defined as *p* < 0.05. (*p < 0.05, **p < 0.01, ***p < 0.001)

To further validate the above inference, BMDMs from *Slc1a5*^*fl/fl*^*Lyz2*^*cre*^ and *Slc1a5*^*fl/fl*^*Lyz2*^*wt*^ mice were isolated and cultured until mature for in vitro experiments (Fig. 3a). LPS was used to stimulate mature BMDMs for subsequent phenotypic analysis. SA-β-gal staining is a classic method for detecting cellular senescence in vitro. Results showed that *Slc1a5* deficiency significantly increased SA-β-gal positive senescent cells in vitro (Fig. 3b-c). Flow cytometry detection of CD86 and CD206 revealed that *Slc1a5* deficient macrophages tend to polarize towards pro-inflammatory phenotype rather than the pro-regenerative phenotype (Fig. 3d-g). PCR detection of cytokines also showed knockout macrophage product increased pro-inflammatory cytokines *Tnf-α*, *Il-1β* and *Il-6*, as well as decreased anti-inflammatory cytokines *Il-10* (Fig. 3h-k). The regenerative factors, *Tgf-β*, *Egf*, *Hgf* and *Pdgf* mentioned and detected previously in vivo were also detected in macrophages via PCR (Fig. 3l-o). Consistent with the in vivo results, regenerative factors in BMDMs are significantly reduced at the transcriptional level. The possible signaling for glutamine deficiency leading to reduced regeneration factors is mTOR signaling [32], which was detected by WB blot. The results showed that both mTOR and phosphorylated mTOR expression were reduced in *Slc1a5* deficient BMDMs (Fig. 3p-r). The above results suggest that *Slc1a5* deficiency promotes macrophage senescence and a pro-inflammatory phenotype, while inhibiting the secretion of their intrinsic pro-regenerative factors.

### *Slc1a5* mediated *Gas6* signaling crucial for macrophage efferocytosis and liver regeneration

**Fig. 4:**
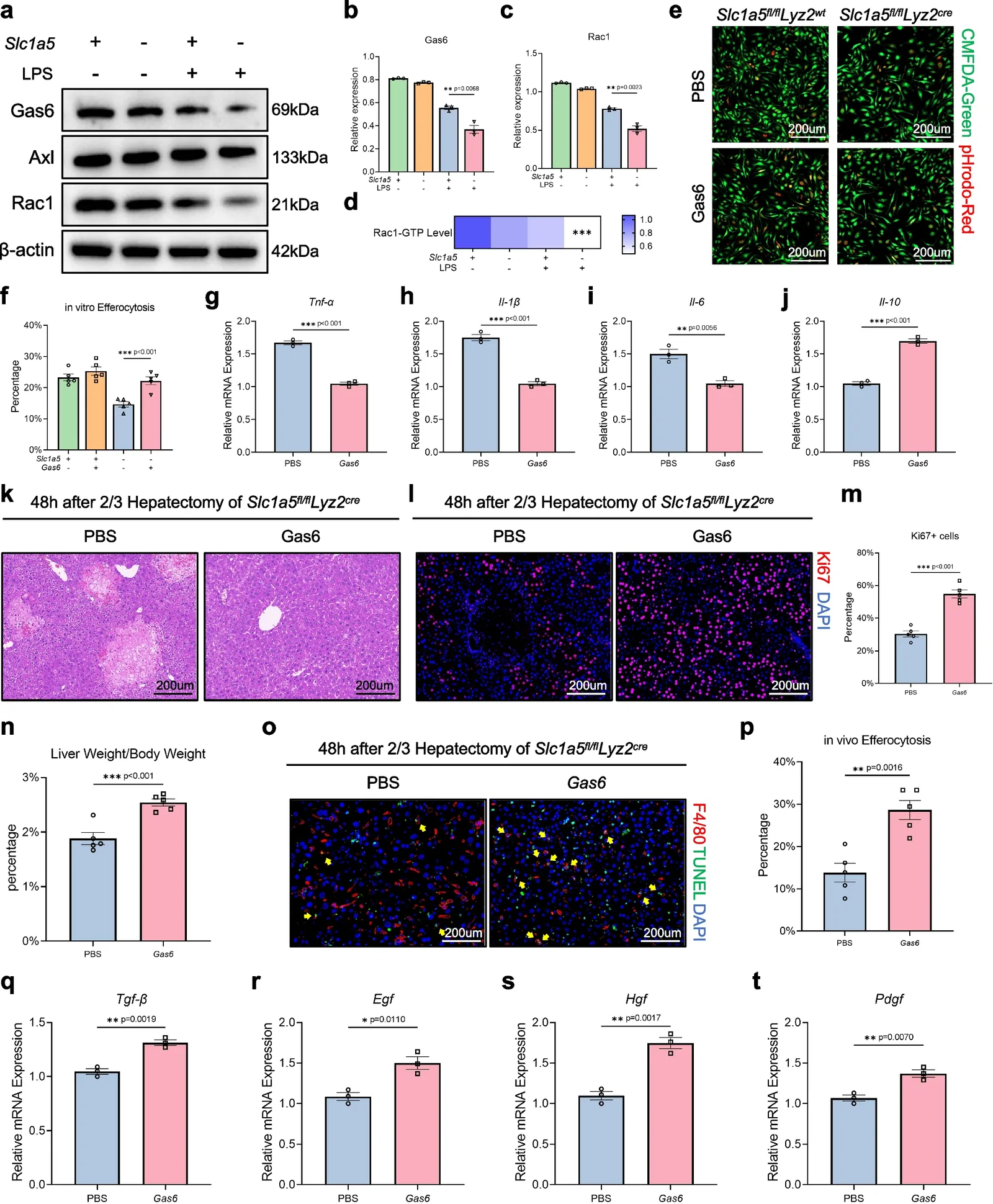
Slc1a5 deficient macrophage impaired liver regeneration via *Gas6* mediated efferocytosis. BMDMs was isolated from *Slc1a5*^*fl/fl*^*Lyz2*^*cre*^ mice and *Slc1a5*^*fl/fl*^*Lyz2*^*wt*^ mice and processed as methods described. LPS was used to stimulate BMDMs. (*n* = 3/group). **a** Western Blot analysis of BMDMs Gas6, Axl and Rac1 expression. **b**-**c** Statistics analysis of Gas6 and Rac1 expression relative to β-actin. **d** Heatmap of Rac1-GPT level detected with G-LISA. Gas6 was used to treat BMDMs (**e**) BMDMs efferocytosis was detected by immunofluorescence photography (scale bar: 200 μm). **f** Statistics analysis of pHrodo-red and CMFDA-green double positive cells in CMFDA-green positive cells to measure in vitro efferocytosis percentage. **g**-**j** PCR analysis of *Tnf-α*, *Il-1β*, *Il-6* and *Il-10* in *Gas6* treated BMDMs. *Slc1a5*^*fl/fl*^*Lyz2*.^*cre*^ mice were subjected to 2/3 hepatectomy model as methods described. Gas6 protein was used before modeling. (*n* = 5/group). **k** Representative H&E staining of liver section (scale bar: 200 μm). **l** Representative Ki67 staining of liver section (scale bar: 200 μm). **m** Statistics analysis of Ki67 positive cells percentage in liver. **n** Statistics analysis of liver weight/body weight ratio. **o** Representative TUNEL and *F4/80* staining of liver section. Efferocytotic macrophages are indicated by yellow arrows. (scale bar: 200 μm). **p** Statistics analysis of F4/80^+^ cells adjacent to TUNEL^+^ cells in all F4/80^+^ cells to measure in vivo efferocytosis. **q**-**t** PCR analysis of *Tgf-β*, *Egf*, *Hgf* and *Pdgf* in liver. Statistical significance was defined as *p* < 0.05. (*p < 0.05, **p < 0.01, ***p < 0.001)

Efferocytosis is a vital function of macrophages, involving the engulfment of dying cells, in tissue regeneration [33]. This process requires ‘find me signal’ receptors, such as the TAM receptor family including AXL, to recognize ‘eat me signal’ [34]. In previous results, we found that *Axl* expression was increased in *Slc1a5* deficient macrophages, whereas *Gas6*, a bridging ligand for efferocytosis, was significantly reduced at transcriptional level. Western Blot confirmed decrease of Gas6 and Rac1 (Rac Family Small GTPase 1), downstream protein of efferocytosis, but not significant change of Axl at translation level (Fig. 4a-c). During the efferocytosis progress, the GTPase activity of downstream efferocytosis signal Rac1 must be activated to allow it to regulate the cytoskeleton and complete the phagocytic process. Therefore, G-LISA was used to detect Rac1 GTPase levels [35]. The results showed that in *Slc1a5* deficient macrophages, *Rac1* activity was also significantly reduced (Fig. 4d). Therefore, it is speculated that *Slc1a5* deficiency primarily impairs macrophage efferocytosis via Gas6. To verify this, Gas6 protein or PBS control was added in supernatant and efferocytosis detection was conducted in vitro in *Slc1a5* deficient or WT BMDMs. It is showed that Gas6 recovered efferocytosis function of *Slc1a5* deficient macrophages (Fig. 4e-f). Besides, *Gas6* inhibited macrophage production of pro-inflammatory *Tnf-α*, *Il-1β* and *Il-6* and increased anti-inflammatory *Il-10* (Fig. 4g-j). Administration of Gas6 in vivo also showed recovery of liver regeneration. HE staining shows that Gas6 accelerates liver tissue regeneration (Fig. 4k). Ki-67 staining revealed that Gas6 significantly increased intrahepatic proliferative cells (Fig. 4l-m). Liver weight/body weight ratio also yielded results of accelerated liver regeneration (Fig. 4n). Recovery of macrophage efferocytosis in vivo after Gas6 administration was also observed through co-staining with F4/80 and TUNEL (Fig. 4o-p). The in vivo expression of regenerative factors *Tgf-β*, *Egf*, *Hgf* and *Pdgf* was also increased by *Gas6* (Fig. 4q-t). The results above suggest that macrophage *Slc1a5* benefits liver regeneration by maintaining efferocytosis via Gas6. Gas6 supplementation in *Slc1a5* deficient mice can restore efferocytosis and improve liver tissue regeneration.

### Macrophage senescence drives impaired efferocytosis and pro-inflammation phenotype

**Fig. 5:**
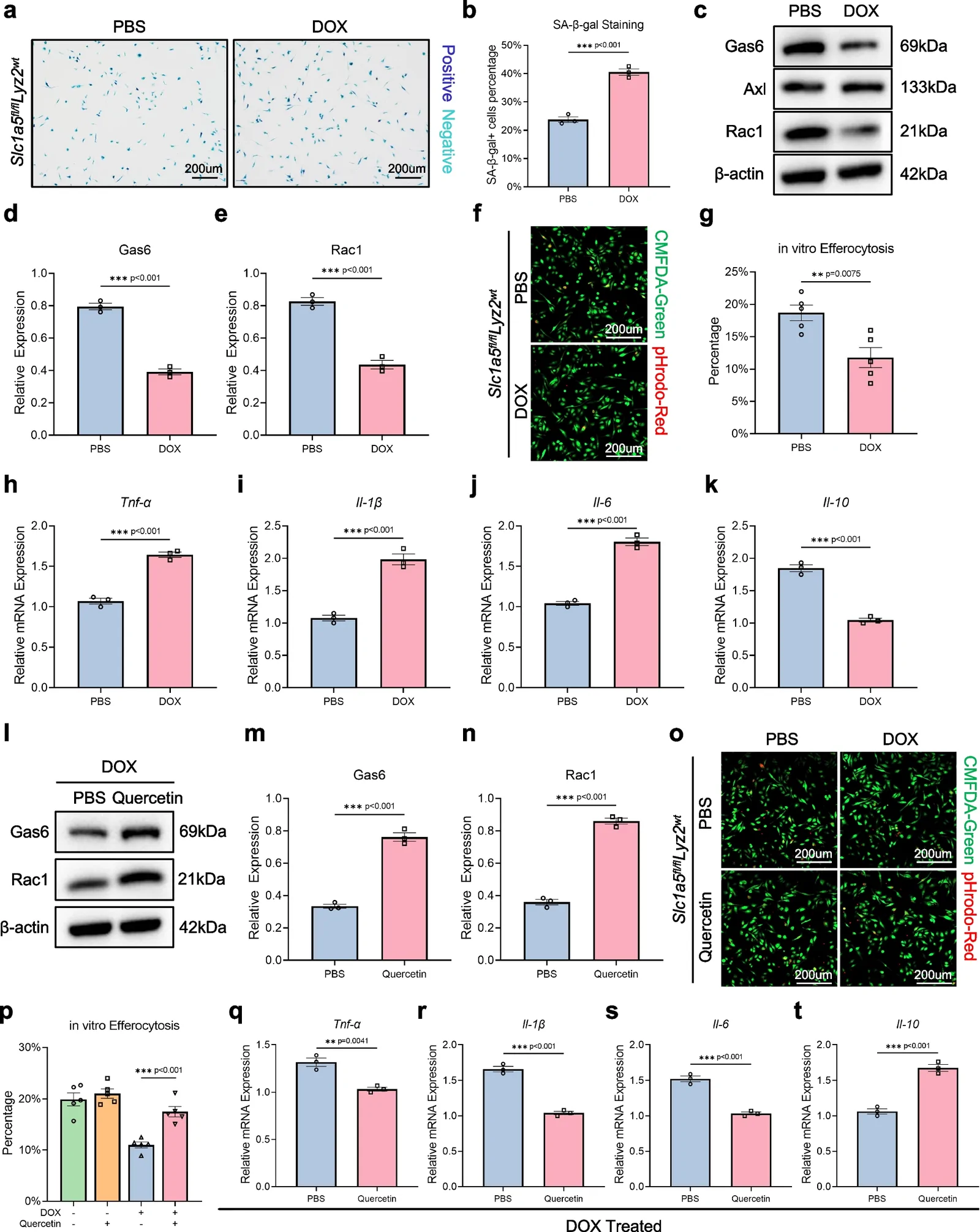
Macrophage senescence drove defective efferocytosis and pro-inflammatory activation. BMDMs was isolated from *Slc1a5*^*fl/fl*^*Lyz2*.^*wt*^ mice and processed as methods described. Doxorubicin was used to induce BMDMs senescence. (*n* = 3/group). **a** Representative SA-β-gal staining of BMDMs. **b** Statistics analysis of SA-β-gal positive cells percentage of BMDMs. **c** Western Blot analysis of BMDMs Gas6, Axl and Rac1 expression. **d**-**e** Statistics analysis of Gas6 and Rac1 expression relative to β-actin. **f** BMDMs efferocytosis was detected by immunofluorescence photography (scale bar: 200 μm). **g** Statistics analysis of pHrodo-red and CMFDA-green double positive cells in CMFDA-green positive cells to measure in vitro efferocytosis percentage. **h**–**k** PCR analysis of *Tnf-α*, *Il-1β*, *Il-6* and *Il-10* in *Gas6* treated BMDMs. Senelytics Quercetin or PBS control was used prevent senescence (*n* = 3/group). **l** Western Blot analysis of BMDMs Gas6 and Rac1 expression. **m**–**n** Statistics analysis of Gas6 and Rac1 expression relative to β-actin. **o** BMDMs efferocytosis was detected by immunofluorescence photography (scale bar: 200 μm). **p** Statistics analysis of pHrodo-red and CMFDA-green double positive cells in CMFDA-green positive cells to measure in vitro efferocytosis percentage. **q**-**t** PCR analysis of *Tgf-β*, *Egf*, *Hgf* and *Pdgf* in liver. Statistical significance was defined as *p* < 0.05. (*p < 0.05, **p < 0.01, ***p < 0.001)

Given that *Slc1a5* deficient macrophages undergo senescence and exhibit impaired efferocytosis, it is necessary to investigate whether this impairment in efferocytosis is driven by cellular senescence. Doxorubicin (DOX), a widely used chemotherapeutic agent known to trigger cellular senescence primarily through the induction of DNA breaks, was employed to stimulate WT BMDMs. SA-β-gal staining confirmed DOX induced macrophage senescence (Fig. 5a-b). Western blot analysis also revealed a decrease in Gas6 and Rac1 under DOX stimulation, consistent with the changes observed in *Slc1a5* deficient BMDMs (Fig. 5c-e). Efferocytosis detection in vitro also showed impairment in DOX treated BMDMs (Fig. 5f-g). Cytokines expression detection also showed pro-inflammatory *Tnf-α*, *Il-1β* and *Il-6* enhancement, as well as anti-inflammatory *Il-10* diminution in senescent macrophages (Fig. 5h-k). Thus, it can be inferred that macrophage senescence impairs efferocytosis function and promotes a pro-inflammatory phenotype.

To perform a rescue experiment for validation, senolytic Quercetin was used in DOX induced senescent macrophages, attempting to reduce the occurrence of senescence. The restoration of Gas6 and Rac1 expression was observed in Quercetin treated BMDMs (Fig. 5l-n). Efferocytosis detection showed that Quercetin significantly restored the DOX-induced impairment of efferocytosis (Fig. 5o-p). In addition, quercetin also alleviated the pro-inflammatory phenotype induced by DOX in BMDMs, as evidenced by the downregulation of pro-inflammatory cytokine *Tnf-α*, *Il-1β* and *Il-6* and upregulation of anti-inflammatory *Il-10* expression (Fig. 5q-t). In other words, alleviating BMDMs senescence with Quercetin helps restore their efferocytosis function and reverse the shift toward a pro-inflammatory phenotype.

### L-Glutamine reverses cell senescence and impaired efferocytosis in SLC1A5 deficiency macrophage

**Fig.6:**
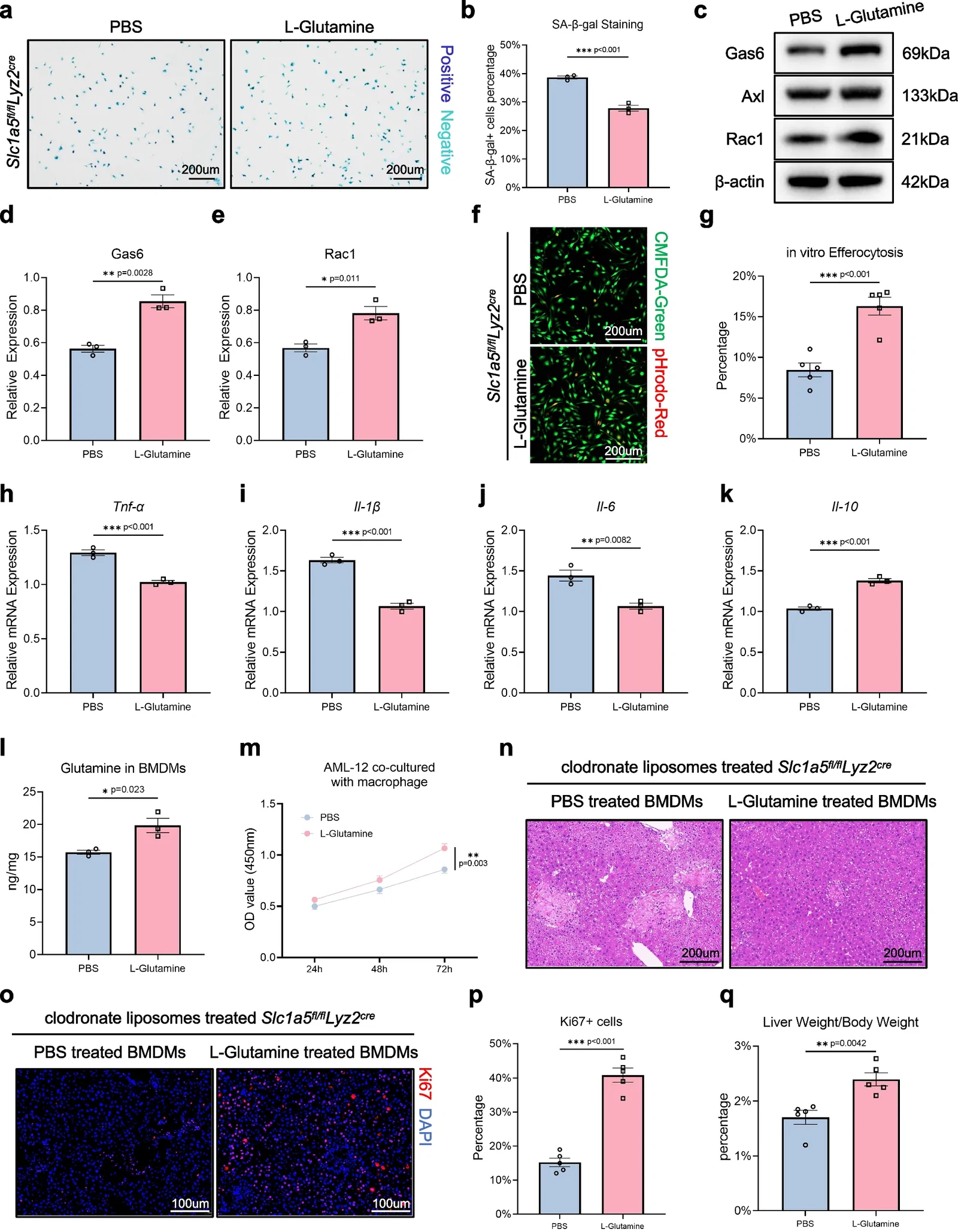
L-Glutamine alleviates Slc1a5 deficient macrophages senescence to restore efferocytosis and attenuate inflammation. BMDMs was isolated from *Slc1a5*^*fl/fl*^*Lyz2*^*cre*^ mice and processed as methods described. L-Glutamine was used to treat BMDMs. (*n* = 3/group). **a** Representative SA-β-gal staining of BMDMs. **b** Statistics analysis of SA-β-gal positive cells percentage of BMDMs. **c** Western Blot analysis of BMDMs Gas6, Axl and Rac1 expression. **d**-**e** Statistics analysis of Gas6 and Rac1 expression relative to β-actin. **f** BMDMs efferocytosis was detected by immunofluorescence photography (scale bar: 200 μm). **g** Statistics analysis of pHrodo-red and CMFDA-green double positive cells in CMFDA-green positive cells to measure in vitro efferocytosis percentage. **h**–**k** PCR analysis of *Tnf-α*, *Il-1β*, *Il-6* and *Il-10* in *Gas6* treated BMDMs. **l** L-Glutamine treated BMDMs glutamine levels in vitro were detected by LC–MS/MS. **m** AML-12 was co-cultured with L-glutamine or PBS control treated macrophage through transwell chamber. The CCK-8 assay is used to detect AML-12 proliferation. *Slc1a5*^*fl/fl*^*Lyz2*.^*cre*^ mice pretreated with clodronate to deplete intrahepatic macrophages were subjected to 2/3 hepatectomy mode. L-Glutamine or PBS treated *Slc1a5* deficient BMDMs were adoptively transferred after modeling. **n** Representative H&E staining of liver section (scale bar: 200 μm). **o** Representative Ki67 staining of liver section (scale bar: 200 μm). **p** Statistics analysis of Ki67 positive cells percentage in liver. **q** Statistics analysis of liver weight/body weight ratio. Statistical significance was defined as *p* < 0.05. (*p < 0.05, **p < 0.01, ***p < 0.001)

SLC1A5 is a high-affinity glutamine transport protein. Therefore, the functional changes in macrophages mediated by *Slc1a5 * deficiency are highly likely driven by glutamine deficiency. *Slc1a5* deficient BMDMs were treated with supplementary glutamine to verify current hypothesis. SA-β-gal staining revealed that macrophage senescence is significantly reduced by glutamine treatment (Fig. 6a-b). With L-Glutamine supplementation, Gas6 and Rac1 level of *Slc1a5* deficient BMDMs is increased in Western Blot detection (Fig. 6c-e). The efferocytosis detection also showed that L-Glutamine supplementation significantly restored the efferocytosis function of *Slc1a5*^*fl/fl*^*Lyz2*^*cre*^ BMDMs. (Fig. 6f-g). Glutamine also decreased pro-inflammatory cytokines *Tnf-α*, *Il-1β* and *Il-6* production and increased anti-inflammatory *Il-10* (Fig. 6h-k). Glutamine level rise in L-Glutamine treated *Slc1a5*^*fl/fl*^*Lyz2*^*cre*^ BMDMs was verified with LC–MS/MS (Fig. 6l).To investigate whether inhibiting macrophage senescence by L-Glutamine promotes hepatocyte proliferation, a co-culture system of AML-12 and BMDMs was established. CCK-8 assay revealed that L-glutamine treated BMDMs significantly promoted the proliferation of co-cultured AML-12 cells (Fig. 6m). For further validation in vivo, L-Glutamine or PBS treated *Slc1a5* deficient BMDMs were adoptively transferred into *Slc1a5*^*fl/fl*^*Lyz2*^*cre*^ mice pretreated with clodronate to deplete intrahepatic macrophages. HE staining showed that L-Glutamine pretreated BMDMs accelerated liver regeneration (Fig. 6n). Ki67 staining also showed an increase in intrahepatic regenerative cells, although it may not have reached normal levels (Fig. 6o-p). Similar results can be observed in liver weight/body weight ratio (Fig. 6q). The results of in vivo and in vitro experiments confirmed that alleviating BMDM senescence with L-Glutamine can mitigate the delay in liver regeneration, which means Slc1a5 mediated glutamine transportation in macrophages is vital in liver regeneration.

### In vivoL-Glutamine administration as a potential therapeutic strategy for liver regeneration

**Fig.7:**
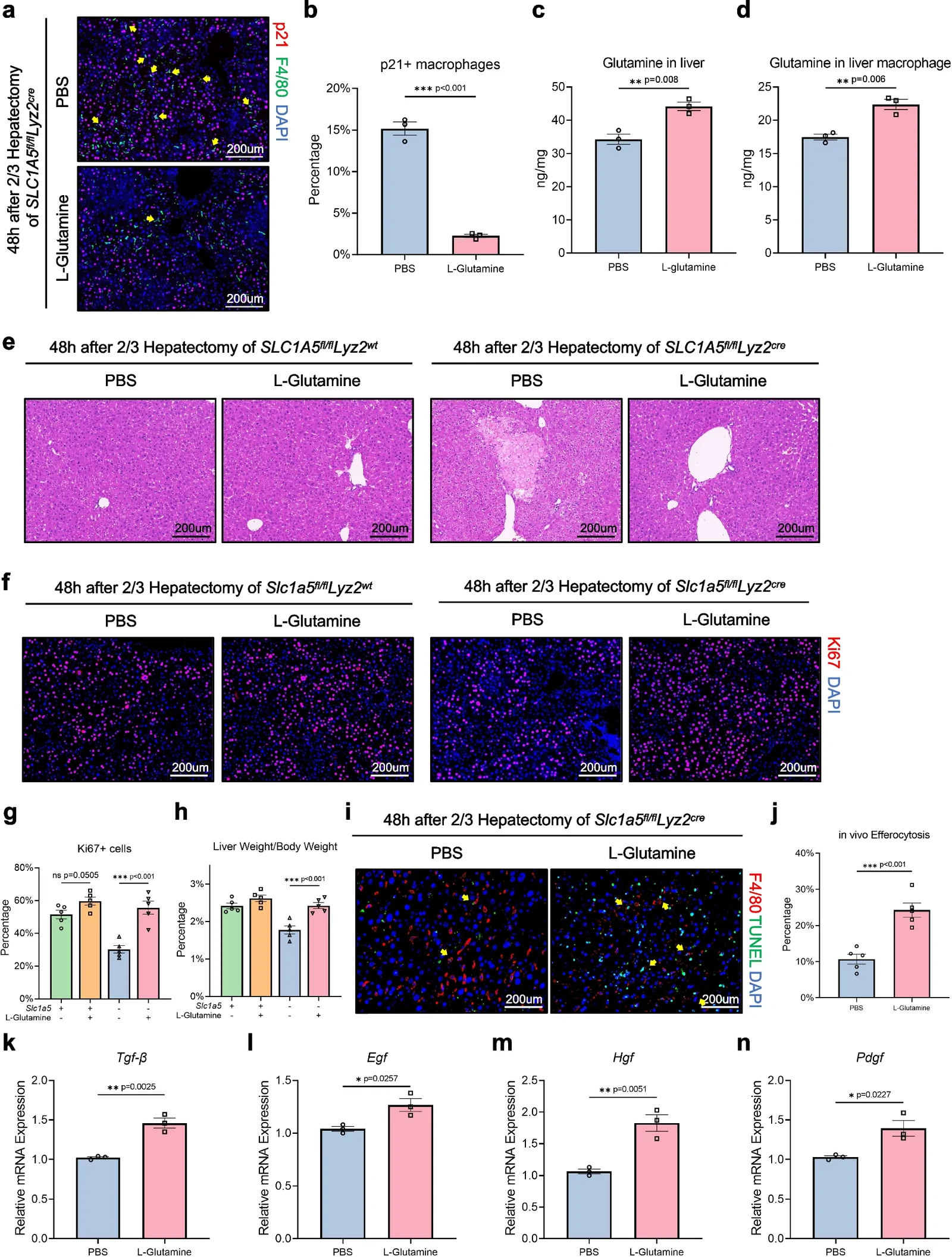
In vivo L-Glutamine administration reduced senescent macrophages to rescue liver regeneration. Mice were subjected to 2/3 hepatectomy model as methods described. L-Glutamine was used before modeling. (*n* = 5/group). **a** Representative p21 and F4/80 staining of liver section. Double-positive cells are indicated by yellow arrows. (scale bar: 200 μm). **b** Statistics analysis of p21 and F4/80 double positive cells in F4/80 positive cells. **c** Liver tissue and (**d**) intrahepatic macrophages glutamine levels in vivo were detected by LC–MS/MS. **e** Representative H&E staining of liver section (scale bar: 200 μm). **f** Representative Ki67 staining of liver section (scale bar: 200 μm). **g** Statistics analysis of Ki67 positive cells percentage in liver. **h** Statistics analysis of liver weight/body weight ratio. **i** Representative TUNEL and F4/80 staining of liver section. Efferocytotic macrophages are indicated by yellow arrows. (scale bar: 200 μm). **j** Statistics analysis of F4/80^+^ cells adjacent to TUNEL^+^ cells in all F4/80^+^ cells to measure in vivo efferocytosis. **k**-**n** PCR analysis of *Tgf-β*, *Egf*, *Hgf* and *Pdgf* in liver. Statistical significance was defined as *p* < 0.05. (*p < 0.05, **p < 0.01, ***p < 0.001)

To further validate the effects of direct in vivo application of L-Glutamine and assess its potential for clinical translation, mice that underwent hepatectomy were supplemented with L-Glutamine by intragastric administration. Likewise, in vivo administration of glutamine markedly decreased senescent macrophages within the liver by F4/80 and p21 co-staining (Fig. 7a-b). Enhanced glutamine level in both liver tissue and intrahepatic macrophage was confirmed with LC–MS/MS (Fig. 7c-d). HE staining revealed accelerated liver regeneration in *Slc1a5*^*fl/fl*^*Lyz2*^*cre*^ mice, whereas no significant difference was observed in *Slc1a5*^*fl/fl*^*Lyz2*^*wt*^ mice (Fig. 7e). More regenerative cells can be observed under the treatment of L-Glutamine in *Slc1a5*^*fl/fl*^*Lyz2*^*cre*^ mice (Fig. 7f-g). Interestingly, Ki67 staining and quantification showed that L-Glutamine treatment also improved cell proliferation in WT mice, reaching marginal significance (*p* = 0.0505). However, this slight proliferative boost did not translate into a significant difference in the liver weight/body weight ratio (Fig. 7h), possibly because the WT liver exhibits a ‘ceiling effect’. Similarly to the Gas6 in vivo administration, F4/80 and TUNEL co-staining revealed that L-Glutamine improved intrahepatic macrophage efferocytosis in *Slc1a5*^*fl/fl*^*Lyz2*^*cre*^ liver (Fig. 7i-j). Furthermore, elevation of intrahepatic regenerative factors *Tgf-β*, *Egf*, *Hgf* and *Pdgf* was also detected by PCR (Fig. 7k-n). These results suggest that in vivo supplementation with L-glutamine can alleviate delay of liver regeneration caused by macrophage senescence, and also point to a potential clinical strategy.

## Discussion

Liver regenerative capacity is important for maintenance of homeostasis of human body. During liver regeneration, almost all hepatocytes are mobilized, indicating that hepatocytes have extensive proliferative activity [36]. Both multilineage tracing and proliferation tracing techniques have confirmed that hepatocytes in the midlobular zone are the main source of newly generated hepatocytes in the adult liver [37, 38]. Macrophages play an important role in liver regeneration. When acute or chronic liver injury leads to macrophage depletion, liver regeneration is severely impaired [39, 40]. Studies have found that an increase in the number of M2 macrophages is associated with liver regeneration, and exogenous supplementation with M2 macrophages alleviates post-hepatectomy liver dysfunction and promotes hepatocyte proliferation [41]. With advances in new technologies such as single-cell omics and spatial transcriptomics, the diversity and dynamics of macrophages are beginning to be recognized. Different KC and MDM subpopulations may play distinct roles in liver regeneration [42–44]. In addition, other non-parenchymal cells (such as HSCs and LSECs) also play important roles in liver regeneration, for example by secreting regenerative factors [45, 46].

Glutamine is a crucial compound for cell metabolism, which affects cell proliferation and senescence, via different signaling molecules like AMPK and mTOR [18, 47]. In our current study, we identified that macrophage *Slc1a5*, an amino acid transporter protein with glutamine as its preferred substrate, was significantly up regulated during liver regeneration. Using *Slc1a5* specific deficiency mice, we found that macrophage *Slc1a5* knockout impaired liver regeneration, which may be related to senescence. In stem-like cancer cells, SLC1A5 determined glutamine-dependent senescence tolerance [48]. Similarly, Inhibition of SLC1A5 induced hepatic stellate cells (HSCs) senescence in fibrotic livers [49]. The enhanced glutamine metabolism induced by calorie restriction confers the body with better resistance to the increased oxidative stress encountered in old age, in which AMPK signaling plays vital role [50]. Glutaminolysis also activated AMPK-MTORC1 signaling axis, which is a key regulator of cell proliferation and cell senescence [51]. Glutamine promote macrophages metabolism reprogramming and anti-inflammatory polarization [52]. We found that *Slc1a5* deficiency accelerate macrophage senescence in vivo and in vitro, and promote macrophage polarize towards an inflammatory phenotype rather than a regenerative one.

Cell senescence is a state of cells, in which cells exit from cell cycle in response to stress and damage, manifest as SASP secretory and metabolic reprogramming [53]. It is noteworthy that unique physiological functions of different cells make their senescence play different roles in the liver regeneration. For example, senescent HSC was reported to stimulate hepatocyte proliferation through the STAT3-YAP pathway and ERK1/2 pathway, respectively, by secreting IL-6 and CXCR2 ligands [54]. When it comes to macrophage, the situation is different. During aging or disease, macrophages undergo changes in metabolism and inflammation regulation. Also, senescent macrophages aggravate most diseases by various ways, including SASP expression [17, 55]. In our study, a significant increase in F4/80^+^/p21^+^ cells within the liver in *Slc1a5*^*fl/fl*^*Lyz2*^*cre*^ mice confirms macrophage senescence and these senescent cells exhibit a significant pro-inflammatory phenotype and reduced secretion of regenerative factors. In in vitro experiments, DOX was used to induce senescence, while Quercetin was used for rescue. As a classic senolytic, Quercetin may combat senescence through multiple pathways or targets, such as the regulation of the cell cycle via *Sirtuin 1*, [56] as well as the modulation of metabolism and inflammation through pathways such as AMPK pathway [57]. In-depth future research into the specific effects of Quercetin on macrophages may play a role in various senescence-related diseases.

It is reported that macrophage efferocytosis function is impaired during aging and related to cell senescence [58, 59]. Interestingly, in the early stage of development, macrophage efferocytosis and subsequent reprogramming is vital for tissue correctly regeneration [12]. In this study, we found that senescent macrophage efferocytosis was impaired due to the decrease of ligand Gas6, while receptor Axl showed a reactive increase in transcription level, but not the protein level, possibly due to a compensatory feedback mechanism. Meanwhile, supplement of Gas6 could reverse the adverse phenotype of macrophages, which is similar to previous reports [60]. Efferocytosis is essential for maintaining homeostasis, as it can eliminate dying cells. What is more, macrophage phenotype transformation after efferocytosis is crucial for disease [61, 62]. Efferocytosis no only reduce DAMPs via removing dying cells, but also increase macrophage M2 polarization and promote tissue regeneration [63–65]. In fact, this is a complex mechanism involving multiple signaling pathways. Firstly, when efferocytosis is insufficient, apoptotic cells undergo secondary necrosis, releasing DAMPs, which promotes the pro-inflammatory activation of macrophages [66]. After efferocytosis, macrophages can also utilize the ingested metabolites to undergo metabolic reprogramming (such as amino acid metabolism, lipid metabolism, and the tricarboxylic acid cycle) and transform into a pro-repair phenotype, and secreting signaling molecules that promote inflammation resolution and tissue regeneration, such as IL-10. [67, 68] So, we focus on the macrophage efferocytosis and functional change. Cell senescence caused by Slc1a5-mediated glutamine transportation obstruction impaired macrophage efferocytosis and promote inflammatory cytokines expression. Although our model represents an acute, stress-induced senescence, it shares core molecular features with chronic age-related senescence, such as elevated p21 expression, SASP secretion and macrophage efferocytosis impairing [69]. This also suggests that the treatment for macrophage senescence we are studying may also play a role in age-related diseases. In future studies, specifically inducing or eliminating macrophage senescence, or developing macrophage-targeted delivery systems for senolytics, may improve liver regeneration. In fact, relevant research has already been conducted on it such as Alzheimer's disease [70].

Slc1a5 is a neutral amino acid transporter with a stronger affinity for glutamine [71]. As a biological fuel, Glutamine supplies carbon and nitrogen to fuel biosynthesis [72]. It may regulate macrophage function through multiple metabolic pathways. It is reported that secondary metabolite of glutamine α-ketoglutarate supports oxidative phosphorylation, thereby maintaining macrophage anti-inflammatory polarization [73]. It is also found that the inhibition of glutamine-induced glutathione production leads to increased intracellular oxidative stress, which can induce macrophage polarization towards proinflammatory phenotype from anti-inflammatory phenotype [74]. This ability makes it crucial in the process of repair in various kinds of cells [75, 76], as well as different kinds cell senescence, including tumor cells and nontumor cells [18, 48]. The exertion of this ability to promote recovery partially rely on Slc1a5, which explain supplement of glutamine in *Slc1a5* myeloid deficiency mice can also rectify macrophage senescence and promote regeneration. It is noteworthy that glutaminase-1-mediated glutaminolysis has also been found to regulate the unique requirements of cellular detoxification and high-energy cytoskeleton reorganization during macrophage efferocytosis [77]. This serves as a valuable complement to our mechanistic study, suggesting that glutamine may regulate efferocytosis not solely through its modulation of senescence. What is interesting, glutamine uptake depend on Slc1a5 in hepatocytes also promotes liver regeneration [26]. In our current study, in vivo L-Glutamine supplementation improved intrahepatic cell proliferation in both *Slc1a5*^*fl/fl*^*Lyz2*^*cre*^ and *Slc1a5*^*fl/fl*^*Lyz2*^*wt*^ mice. These results suggest that glutamine and SLC1A5 play critical roles in different intrahepatic cells during liver regeneration. This highlights a coordinated, multicellular mechanism driving the regenerative process.

Several limitations of this study should be acknowledged. Firstly, BMDMs senescence was induced in vitro via Doxorubicin, which confirmed by SA-β-gal staining. It must be acknowledged that Doxorubicin is a DNA-damaging agent with broad cytotoxic effects beyond inducing senescence. It would be better incorporating additional senescence inducers, such as γ-irradiation, in the future study. Secondly, the plasticity of macrophages in living tissues is influenced by numerous microenvironmental factors, which in vitro BMDM assays cannot completely recapitulate. Ideally, many assays would be better performed directly on intrahepatic macrophages isolated after hepatectomy. Thirdly, there is a lack of separate assessments for the functions of various intrahepatic cell types during liver regeneration. Future studies leveraging single-cell omics profiling are poised to provide deeper insights into this phenomenon.

In summary, our current study identified the crucial role of macrophage Slc1a5 in transporting glutamine, which regulates cell senescence. Glutamine deficiency induced macrophage senescence inhibit macrophage efferocytosis and subsequent phenotypic transformation via down regulating *Gas6* expression. Suppressed liver regeneration is associated with efferocytosis impaired macrophage and can be reversed by supplementing with additional glutamine. Based on the findings of our study, enhancing glutamine availability in macrophages may represent a promising therapeutic strategy to mitigate macrophage senescence and promote liver regeneration.

## Materials and methods

### Animals

*Slc1a5* myeloid-specific knockout mice (*Slc1a5*^*fl/fl*^*Lyz2*^*cre*^) were generated and bred by GemPharmatech Co. Ltd. (Nanjing, China) on a C57BL/6 background, following a mature strategy: *Slc1a5*^*fl/fl*^ mice (*Slc1a5*, with loxP sites flanking the critical region of the *Slc1a5* gene) was crossed with myeloid-specific Cre (*Lyz2*-Cre) mice to generate F1 heterozygous offspring. Subsequent backcrossing or intercrossing of F1 mice was performed to screen for F2 mice harboring the target genotype. Genotype was conducted via PCR analysis of genomic DNA extracted from mouse tail tips. *Slc1a5*^*fl/fl*^*Lyz2*^*wt*^ mice were treated as wild-type control (WT).

All experimental mice were 8–10 weeks old males. All mice were housed in a standard specific pathogen-free (SPF) environment with regulated light (12 h of light and dark photocycle) and temperature (21 ± 2 °C). Bedding was changed twice weekly, and mice had ad libitum access to standard rodent chow and sterilized drinking water. Detailed welfare monitoring protocols included: daily observation of mental status, mobility, food/water intake, and fur luster; weekly body weight measurement and growth curve recording. All animals received humane care and all animal procedures met the relevant legal and ethical requirements according to the protocols authorized by the Institutional Animal Care and Use Committee of Nanjing Medical University (IACUC-2311005).

### Mouse 2/3 hepatectomy model and specimen processing

A mature model of 2/3 hepatectomy has been used to study liver regeneration [78]. To be brief, after inhalation anesthesia with 2% isoflurane, a midline laparotomy was performed. The left and median lobes were removed after ligation with silk thread, followed by closure of the abdomen. Intraperitoneal injection of carprofen is used for postoperative analgesia. Mice were CO_2_ anesthesia sacrificed in 0 or 48 h (peak of liver regeneration, which is reported previously) [28, 29] after modeling and liver samples were harvest for analysis.

For conducting the rescue experiments, recombinant mouse Gas6 protein (5 μg/mouse [79], R&D system, cat: 986-GS) was injected intraperitoneally 1 h before modelling. For glutamine gavage experiment, mice were treated with L-Glutamine (25 mg/kg [20], MedChemExpress, cat: HY-N0390) by intragastric administration after modelling.

Liver samples were fully immersion fixed in 4% paraformaldehyde (Beyotime, cat: P0099) for at least 48 h and embedded in paraffin for hematoxylin and eosin (H&E) staining and immunofluorescent (IF) staining. Antibody for IF staining is detailed in Supplementary Table 1. Full-slide scanning imaging was scanned and imaged under a 20 × objective lens with a multi-channel fluorescence scanner (3DHISTECH, Pannoramic MIDI), and the images were viewed and processed using SlideViewer software.

### Statistical data analysis

The results are presented as the mean ± SEM. For comparisons between two groups with normal distribution, a two-tailed Student’s t-test was used. For comparisons among multiple groups, a one-way analysis of variance (ANOVA) followed by Tukey’s post-hoc test was performed. All statistical analyses were conducted using GraphPad Prism 8 software and the bar plots were generated. Statistical significance was defined as *p* < 0.05. (*p < 0.05, **p < 0.01, ***p < 0.001).

## Supplementary Information


Supplementary Material 1.

## Data Availability

The data supporting the findings of this study are available within the article and supplementary materials.
