# Supplementary material for: Solute Carrier Family 1 Member 5 (SLC1A5)-dependent glutamine transport regulates macrophage senescence and efferocytosis during liver regeneration
Source: Mol Biomed. 2026 Sep 1;7:155. doi: 10.1186/s43556-026-00526-0 (PMC13534412; doi:10.1186/s43556-026-00526-0)
Supplement: Supplementary file 1 [file 43556_2026_526_MOESM1_ESM.docx]

**Title Page**

**Solute Carrier Family 1 Member 5 (SLC1A5)-dependent glutamine transport regulates macrophage senescence and efferocytosis during liver regeneration**

Haoran Hu^1,2,†^, Tao Han^1,†^, Qiyuan Huang^1,†^, Ping Gong^1,4,†^, Jiangxi Liu^5,†^, Hao Wang^1^, Xiaowei Ling^1^, Kang Wang^1^, Shuangshuang Liu^1^, Yue Yu^1^, Ting Luo^3,*^, Feng Cheng^1,*^ Xiaoxiao Liu^2,3,^^*^

^1^. Hepatobiliary Center, The First Affiliated Hospital with Nanjing Medical University, Research Unit of Liver Transplantation and Transplant Immunology, Key Laboratory of Liver Transplantation, Chinese Academy of Medical Sciences, Key Laboratory of Hepatobiliary Tumors, National Health Commission, Jiangsu Provincial Medical Innovation Center, Jiangsu Provincial Medical Key Laboratory, Nanjing, 210029, China.

^2^. Department of Radiation Oncology, Cancer Center, Affiliated Hospital of Xuzhou Medical University; Cancer Institute, Xuzhou Medical University, Xuzhou, Jiangsu, 221000, China.

^3^. Institute of Breast Health Medicine, Breast Center, Department of Medical Oncology, Cancer Center, West China Hospital, Sichuan University, Chengdu, Sichuan, 610041, China.

^4^. Department of General surgery, People’s Hospiltal of Jinhu, Huaian, Jiangsu, 213200, China.

^5^. Jiangsu Key Laboratory of Organ Transplantation and Transplant Immunology and Hepatobiliary Center, Affiliated Hospital of Xuzhou Medical University, Xuzhou, Jiangsu, 221000, China

^†^. These authors contributed equally to this work.

^*^. Correspondence should be addressed to:

Prof. Xiaoxiao Liu, E-mail: [drliuxx@126.com](mailto:drliuxx@126.com);

Prof. Feng Cheng, E-mail: [docchengfeng@njmu.edu.cn](mailto:docchengfeng@njmu.edu.cn);

Prof. Ting Luo, E-mail: [luoting@wchscu.cn](mailto:luoting@wchscu.cn);

**Supplemental Materials and Methods**

**Isolation and culture of bone marrow derived macrophages (BMDMs)**

Mice femurs and tibias were removed from the euthanized mice. Bone ends were cut off and then marrow cells were flushed out and filtered through a 70-μm strainer (Corning, cat: 352350). Cells were cultured in Dulbecco’s modified Eagle’s medium (DMEM, ThermoFisher Scientific, cat: 11965092) supplemented with 10% Fetal Bovine Serum (FBS, ThermoFisher Scientific, cat: A5669402), 1% Penicillin-Streptomycin (100× P/S, ThermoFisher Scientific, cat: 15140122), 1% HEPES (ThermoFisher Scientific, cat: 15630080) and 20ng/ml macrophage Colony Stimulating Factor (mCSF, ThermoFisher Scientific, cat: 315-02-1MG) in a humidified CO_2_ incubator at 37 °C. The medium was replaced to remove non-adherent cells in day3, and continue culture until day7 to obtain mature macrophage (over 95% F4/80 positive). BMDMs were replated for further experiments.

For mechanism study, 100ng/ml lipopolysaccharides (LPS, MedChemExpress, cat: HY-D1056) was used to activate macrophage for 24 hours. 5μmol/L doxorubicin hydrochloride (DOX, MedChemExpress, cat: HY-15142) was added into medium for 2 hours to induce macrophage senescence. To prove causality, 10umol/L Quercetin (MedChemExpress, cat: No.522-12-3), an acknowledged senelytics, was used to combat DOX-induced senescence. For rescue experiments, GAS6 (100ng/ml) or L-Glutamine (10mmol/L) were added to the culture system to pretreat BMDMs for 12 hours.

**Isolation of primary intrahepatic macrophage via in situ liver perfusion**

The liver was cannulated via the portal vein and initially perfused with 20 ml of Ca^2+^/Mg^2+^ free Hanks’ Balanced Salt Solution (HBSS, ThermoFisher Scientific, cat: 14175095) to flush out the blood, while the inferior vena cava was cut to allow drainage. Subsequently, the liver was perfused with 20 ml of Ca^2+^/Mg^2+^ containing HBSS (ThermoFisher Scientific, cat: 14025092) supplemented with 0.25 mg/mL Collagenase IV (BioFroxx, cat: EZ7890B261) for digestion. The liver was then sacrificed and transfer to new dish with PBS on ice. The tissue was gently genteelly decapsulated, and shaken to release the liver solution containing hepatocytes and liver NPCs. The suspension was filtered through a 70 µm cell strainer and centrifuged at 50× g to remove hepatocytes. The supernatant was collected and centrifuged at 500× g. The resulting pellet was resuspended and purified using a Percoll (GE Healthcare, cat: 10290514) density gradient, consisting of (from top to bottom): the cell suspension layer, a 30% Percoll layer, and a 70% Percoll layer. After centrifugation at 2400 rpm for 30 minutes, the white cell layer at the interface was collected. These cells were washed and cultured in complete DMEM for 2 hours; the adherent cells were collected as intrahepatic macrophages.

**Analysis of single-cell RNA sequencing data**

The liver regeneration data analyzed in this study were obtained from CNGBdb (China National GeneBank DataBase) scRNA-seq datasets (CNP0002310). Raw data were processed using the Seurat package (v4.0.5) in R. Cells with fewer than 200 detected genes or with mitochondrial gene content greater than 10% were excluded. DoubletFinder was applied to identify and remove doublets. The filtered gene expression matrix was normalized using the ‘LogNormalize’ method, and variable genes were identified for downstream analysis. We utilized the Harmony algorithm for dataset integration to correct for batch effects. Then, we selected the top 3,000 highly variable genes (HVGs) using the ‘vst’ method in Seurat and used the top 30 principal components for downstream clustering and visualization.

Dimensionality reduction was conducted using principal component analysis (PCA), followed by clustering using the Louvain algorithm and visualization via Uniform Manifold Approximation and Projection (UMAP). Cell types were annotated based on canonical marker genes.

To examine the expression dynamics of *Slc1a5*, we generated dot plots to visualize expression across different cell types and time points, and violin plots to assess its distribution within individual cell types.

**BMDMs RNA sequencing and analysis**

RNA sequencing was performed using BMDMs from *SLC1A5^fl/fl^Lyz2^cre^* mice or *SLC1A5^fl/fl^Lyz2^wt^* mice as individual biological replicates (n = 3 mice/group). Total RNA was extracted with TRIzol reagent (ThermoFisher Scientific, cat: 15596026CN). RNA quality was assessed, and samples with RIN values > 7.0 were used. mRNA library preparation and RNA-seq was performed by Novogene using Illumina Novaseq platform with reads at a depth of approximately 40 million reads per sample. Differential expression analysis was performed using the DESeq2 R package (1.20.0). Genes with an adjusted P-value < 0.05 and absolute foldchange >2 found by DESeq2 were assigned as differentially expressed. Gene Ontology (GO) enrichment analysis of differentially expressed genes was implemented by the clusterProfiler R package, in which gene length bias was corrected. GO terms with corrected P-value < 0.05 were considered significantly enriched by differential expressed genes. The data reported in this paper have been deposited in the OMIX, China National Center for Bioinformation (https://ngdc.cncb.ac.cn/omix: accession no. OMIX011223).

**Cell senescence detection (SA-β-gal staining)**

Macrophage senescence was measured with Senescence β-Galactosidase Staining Kit (Beyotime, cat: C0602) according to the manufacturer’s protocols. In brief, the cells in the six-well plate were fixed with 1ml of fixative for 15 minutes, washed three times with PBS, and then added with 1ml of prepared staining working solution. The plate was sealed with parafilm and stained for 24 hours under the condition of pH=6.0 (titrated with dilute hydrochloric acid). After washing with 70% ethanol to remove crystals, 2ml of PBS was added for observation. Image was obtained by Olympus optical microscope with BioHD-C20 camera. Cells that have been dyed dark blue are determined to be positive, while those in light blue are considered negative. To ensure objectivity, cell counting was conducted manually by two independent observers blinded to the experimental conditions.

In vivo macrophage senescence detection was measured by co-staining of F4/80 and p21. F4/80 and p21 double positive cells are identified as senescent macrophages.

**In vitro and in vivo efferocytosis detection**

*In vitro* efferocytosis detection was conducted as previous reported. BMDMs were pre-stained with 1μmol/L CMFDA-Green (Yeason Biotechnology, cat: 40721ES50). PHrodo-Red (ThermoFisher Scientific, cat: P36600) labeled apoptotic AML-12 cells (induced with Apoptosis Inducer Kit (Beyotime, cat: c0006s) for 12 hours) were incubated with BMDMs for 45 minutes at a ratio of 5:1. Efferocytosis was detected with a Zeiss LSM 900 laser scanning Confocal Microscopy. Taking into account the characteristic of pHrodo emitting strong fluorescence under acidic conditions (when engulfed into lysosomes), the evaluation of efferocytosis is defined as pHrodo-Red & CMFDA-Green double-positive cells / CMFDA-Green positive cells.

In vivo efferocytosis detection was measured by co-staining of F4/80 and TUNEL in liver section. F4/80+ macrophages immediately adjacent to TUNEL+ cells are believed to be undergoing efferocytosis.

***Rac1* activity G-LISA assay**

BMDMs underwent efferocytosis were washed with PBS to clear dead cells and subjected to a Rac1 G-LISA GTPase activation assay (Cytoskeleton; cat: BK128) according to the manufacturer’s instructions. The signal is read by measuring absorbance at 490 nm using a microplate spectrophotometer.

**Flow cytometry for macrophage inflammatory phenotype**

Pretreated BMDMs were collected and washed by PBS. After blocking with TruStain FcX PLUS (anti-mouse CD16/32, Biolegend, cat: 156604) for 10 minutes on 4 °C. Cells were incubated with the indicated fluorescence-labeled antibodies for 20 minutes on 4 °C for extracellular staining and after fixation and permeabilization, stain for 20 minutes to perform intracellular staining protected from light on 4 °C (Supplementary Table 1). And then acquired on a BD FACSCelesta™ Multicolor Flow Cytometer. Fluorescence compensation was calculated using single-stained controls. Cells were gated on FSC-A/SSC-A, followed by doublet exclusion (Singlets) using FSC-A/FSC-H. Macrophages were subsequently identified as F4/80 positive cells. Target marker expression was analyzed within the F4/80 positive parent gate. CD86-positive CD206-negative cells were identified as pro-inflammatory polarization, while CD86-negative CD206-positive cells are identified as anti-inflammatory polarization. Data were analyzed with FlowJo v10.6.2. Expression levels were determined by Median Fluorescence Intensity (MFI).

**Detection of Glutamine via LC-MS/MS**

LC-MS/MS was used to detect glutamine level in liver sample, primary liver macrophage and BMDMs entrusted to Jiangbei New Area Biopharmaceutical Public Service Platform Co., Ltd. using a Thermo Scientific™ Vanquish™ UHPLC system coupled to a TSQ Quantis™ triple quadrupole mass spectrometer. Briefly, 50 µL of sample homogenate was mixed with 200 µL of extraction solvent (acetonitrile: methanol = 1:1, v/v) containing isotopically labeled internal standards. The mixture was vortexed for 30 s, sonicated for 10 min in an ice-water bath, and incubated at -40°C for 1 h to precipitate proteins. After centrifugation at 12,000 rpm for 15 min at 4°C, the supernatant was collected for analysis. Chromatographic separation was performed on a Waters ACQUITY UPLC BEH Amide column using a gradient elution. Mobile phase A consisted of water containing 25 mM ammonium formate and 0.1% formic acid, and mobile phase B was acetonitrile containing 0.1% formic acid. The mass spectrometer was operated in positive electrospray ionization (ESI+) mode with Multiple Reaction Monitoring (MRM). Data acquisition and processing were performed using TraceFinder software. The concentration of glutamine was determined using calibration curves constructed from authentic standards and normalized to the internal standard signal.

**Hepatocyte proliferation detection (CCK-8 assay)**

To evaluate the role of macrophages in hepatocyte regeneration, hepatocyte cell line AML-12 was co-cultured with L-glutamine or PBS control treated macrophage through transwell chamber in 24-well plates. The CCK-8 assay is used to detect the proliferation of AML-12. Approximately 2500 AML-12 cells in 300ul per well were seeded into 24-well plates. Approximately 1000 macrophages were added on transwell chamber. Before the detection, the cell chamber containing macrophages was removed. Next, 30μL of the CCK-8 reagent (Beyotime, cat: C0038) was added to each well, and the microplate was incubated for 2h at 37°C. The OD450 was measured using a microplate reader (Multiskan Sky) every 24h for 72h.

**Western Blot**

Tissues and cellular proteins were extracted using Radio Immunoprecipitation Assay (RIPA) Lysis buffer (Cell Signaling Technology, cat: 9806) supplemented with protease and phosphatase inhibitors cocktail (Cell Signaling Technology, cat: 5872). For tissue samples, tissues were immersed in RIPA lysis buffer containing protease and phosphatase inhibitors. Homogenization was performed using a bead mill homogenizer with zirconia beads. The homogenates were then incubated on ice for 30 min to ensure complete lysis.

For cell samples, cell pellets were directly resuspended in the lysis buffer and incubated on ice for 30 min. Following lysis, samples were centrifuged at 12,000rpm for 10 minutes at 4°C. The supernatant was carefully collected, while strictly avoiding the upper lipid layer and the bottom pellet, to obtain the total protein extract. Proteins were subjected to 10% sodium dodecyl sulfate-polyacrylamide gelelectrophoresis (SDS-PAGE) at a constant voltage of 120 V and transferred onto a polyvinylidene 550 fluoride (PVDF) membrane at a constant current of 250 mA in an ice-water bath. The duration of both electrophoresis and transfer was optimized for specific target proteins based on their molecular weights to ensure optimal separation and transfer efficiency. After blocking with Rapid Blocking Buffer (ABclonal, cat: RM02956) for 30 minutes, antibodies against the detection targets (Supplementary Table 1) were used and incubated overnight at 4°C. After 2 h of incubation with the appropriate horseradish peroxidase (HRP)-conjugated secondary antibody, bands were detected with SuperFemto ECL Chemiluminescence Kit (Vazyme Biotechnology, cat: E423-02) using a Vilber chemiluminescent imaging system. We used auto-exposure settings or manually adjusted exposure time to ensure bands were visible but not saturated, allowing for accurate densitometry analysis. Gray value of each band was analyzed with Image J software. Target protein levels were normalized to β-actin. All experiments were performed with at least three independent biological replicates.

**Quantitative real-time PCR**

Total RNA was extracted from frozen liver tissue and cells using TRIzol reagent (ThermoFisher Scientific, cat: 15596026CN) following the manufacturer’s instructions. Subsequently, 500ng of total RNA was reverse-transcribed into complementary DNA (cDNA) using the Transcriptor First Strand cDNA Synthesis Kit (Roche, cat: 04896866001) with anchored-oligo(dT)18 and random hexamers primers mix. qRT‐PCR was carried out with cDNA in triplicate using ChamQ SYBR qPCR Master Mix (Vazyme, cat: Q311-02) on the QuantStudio 5 Real‐Time PCR System. Reactions were performed in technical triplicates for each of the three independent biological replicates. No-template controls (NTC) were included to assess contamination. Primer specificity was confirmed by melt curve analysis, and amplification efficiency was validated to be within 90–110%. Gene expression values were calculated using the 2^−ΔΔCt^ method and normalized against *β-actin*. The primers information is detailed in Supplementary Table 2.

Supplementary Table 1. Dyestuff and Murine antibodies summary

| **Name** | **Usage** | **Company & Catalog** |
| --- | --- | --- |
| TruStain FcX^TM^ PLUS (anti-mouse CD16/32) | Fc | Biolegend, cat:156604 |
| FITC anti-mouse F4/80 | Fc | Biolegend, cat:123108 |
| PerCP anti-mouse CD86 | Fc | Biolegend, cat:105025 |
| Brilliant Violet 421^TM^ anti-mouse CD206 (MMR) | Fc | Biolegend, cat:141717 |
| CMFDA-Green | Efferocytosis Detection | Yeason Biotechnology, cat:40721ES50 |
| PHrodo-red | Efferocytosis Detection | ThermoFisher Scientific, cat:P36600 |
| β-Actin Mouse mAb | WB | ABclonal, cat:AC004 |
| GAS6 Rabbit pAb | WB | ABclonal, cat:A8545 |
| Axl (E3Z2H) Rabbit mAb | WB | Cell Signaling Technology, cat:18554 |
| Rac1/Cdc42 Antibody | WB | Cell Signaling Technology, cat:4651 |
| mTOR (7C10) Rabbit Monoclonal Antibody | WB | Cell Signaling Technology, cat:2983 |
| Phospho-mTOR (Ser2448) (D9C2) Rabbit Monoclonal Antibody | WB | Cell Signaling Technology, cat:5536 |
| Anti-rabbit IgG, HRP-linked Antibody | WB | Cell Signaling Technology, cat:7074 |
| Anti-mouse IgG, HRP-linked Antibody | WB | Cell Signaling Technology, cat:7076 |
| CDKN1A/p21 Rabbit mAb | IF | ABclonal, cat:A19094 |
| Ki67 Rabbit mAb | IF | ABclonal, cat:A20018 |
| Anti-F4/80 antibody | IF | Abcam, cat:ab300421 |
| TSA fluorescence kit | IF | Meladybio, cat:ML0088-01 |
| DAPI | IF | Beyotime, cat:C1002 |

* Fc, Flow Cytometry; WB, Western Blot; IF, Immunofluorescence.

Supplementary Table 2. Detailed primers information

| **Gene** | **Species** | **Sequence (5'-3')** |
| --- | --- | --- |
| *Slc1a5* | Mouse | Forward: CATCAACGACTCTGTTGTAGACC  Reverse: CGCTGGATACAGGATTGGG |
| *Tnf-α* | Mouse | Forward: CAGGCGGTGCCTATGTCTC  Reverse: CGATCACCCCGAAGTTCAGTAG |
| *Il-1β* | Mouse | Forward: TTCAGGCAGGCAGTATCACTC  Reverse: GAAGGTCCACGGGAAAGACAC |
| *Il-6* | Mouse | Forward: TCTATACCACTTCACAAGTCGGA  Reverse: GAATTGCCATTGCACAACTCTTT |
| *Il-10* | Mouse | Forward: CTTACTGACTGGCATGAGGATCA  Reverse: GCAGCTCTAGGAGCATGTGG |
| *Tgf-β* | Mouse | Forward: CCACCTGCAAGACCATCGAC  Reverse: CTGGCGAGCCTTAGTTTGGAC |
| *Egf* | Mouse | Forward: TTAACGGGACAGGACTAGAGAAA  Reverse: AAGGAACTTAGAAGAACTCGGGA |
| *Hgf* | Mouse | Forward: AACAGGGGCTTTACGTTCACT  Reverse: CGTCCCTTTATAGCTGCCTCC |
| *Pdgf* | Mouse | Forward: TGGCTCGAAGTCAGATCCACA  Reverse: TTCTCGGGCACATGGTTAATG |
| *β-actin* | Mouse | Forward: GTGACGTTGACATCCGTAAAGA  Reverse: GCCGGACTCATCGTACTCC |
